# Resonant Grating without a Planar Waveguide Layer as a Refractive Index Sensor

**DOI:** 10.3390/s19133003

**Published:** 2019-07-08

**Authors:** Sivan Isaacs, Ansar Hajoj, Mohammad Abutoama, Alexander Kozlovsky, Erez Golan, Ibrahim Abdulhalim

**Affiliations:** 1 Department of Electrooptics and Photonics Engineering and The Ilse Katz Institute for Nanoscale Science and Technology, School of Electrical and Computer Engineering, Ben Gurion University of the Negev, Beer Sheva 84105, Israel; 2 The Weiss Family Laboratory for Nano-scale Systems, Ben-Gurion University of the Negev, Beer Sheva 84105, Israel

**Keywords:** guided mode resonance, biosensors, gratings sensors, waveguide sensors

## Abstract

Dielectric grating-based sensors are usually based on the guided mode resonance (GMR) obtained using a thin planar waveguide layer (PWL) adjacent to a thin subwavelength grating layer. In this work, we present a detailed investigation of thick subwavelength dielectric grating structures that exhibit reflection resonances above a certain thickness without the need for the waveguide layer, showing great potential for applications in biosensing and tunable filtering. Analytic and numerical results are thoroughly discussed, as well as an experimental demonstration of the structure as a chemical sensor in the SWIR (short wave infrared) spectral range (1200–1800 nm). In comparison to the GMR structure with PWL, the thick grating structure has several unique properties: (i) It gives higher sensitivity when the spaces are filled, with the analyte peaking at certain space values due to an increase in the interaction volume between the analyte and the evanescent optical field between the grating lines; (ii) the TM (transverse magnetic) resonance, in certain cases, provides a better figure of merit; (iii) the sensitivity increases as the grating height increases; (iv) the prediction of the resonance locations based on the effective medium approximation does not give satisfactory results when the grating height is larger than a certain value, and the invalidity becomes more severe as the period increases; (v) a sudden increase in the Q-factor of the resonance occurs at a specific height value accompanied by the high local field enhancement (~10^3^) characteristic of a nano-antenna type pattern. Rigorous numerical simulations of the field distribution are presented to explain the different observed phenomena.

## 1. Introduction

Resonances in grating-based structures were observed for the first time by Wood in 1902 [1], although in metallic gratings not dielectric ones. He observed anomalies in the diffraction efficiency spectrum now known as “Wood anomalies”, which he could not explain by ordinary grating theory. In 1907, Rayleigh suggested an explanation for Wood anomalies [2]. According to Rayleigh, for an incident angle θ_m_ the refractive beam of the m^th^ order diffracted wave becomes tangent to the surface grating before disappearing. In this case, for wavelengths greater than a specific value, defined as the Rayleigh wavelength, this m^th^ order beam becomes evanescent, and its energy is redistributed over the other orders. In 1941, Fano presented another explanation of Wood’s anomalies [3], in which he suggested that the resonances in the diffraction efficiency occur due to coupling between the guided waves and vanishing diffracted modes. With the beginning of the epoch of computers, there has been additional progress in the study of Wood’s anomalies. In 1965, Hessel and Oliner presented the first numerical results that explained the resonant anomalies of the poles and the zeros of the diffraction efficiency [4]. Later, Neviere [5] used a pole-zero approach for structures that included the grating and dielectric waveguide layers in order to predict the maximum value in the reflectance spectrum. Popov et al. [6] also used this pole-zero model in their studies and showed that in a dielectric waveguide, the diffraction efficiency has a maximum in the vicinity of the excited guided waves. Wang et al. [7] drew the ranges of the resonance location, showing possible deviations from the Rayleigh wavelength in certain cases. Recently [8] the bridging pole approach of the reflectivity function was applied and was shown to fit reasonably well with the rigorous calculations.

The interest in optimizing the guided mode resonance (GMR) structure has grown in the last two decades due to the importance of this device as a tunable filter [9,10,11,12,13,14], modulator [15,16], and sensor [17,18,19,20,21,22,23,24,25]. This phenomenon is manifested by a sharp peak in the zero order diffraction spectrum of waveguide gratings. GMR occurs due to the coupling of the externally propagating diffracted fields to the modes of the waveguide. For definite parameters of the grating waveguide structure, the location of the narrow peak in the reflection spectrum depends on the incident angle, polarization, and refractive index of the superstrate. In view of these properties, GMR structures can be used in many different fields, such as biosensing, optoelectronics, optical communications, and more. The majority of the works so far deal with thin dielectric grating coupled to a thin dielectric planar waveguide layer (PWL), and only a few dealt with thick dielectric grating, only without PWL, particularly not as a sensor or filter. The interest in thick gratings, and particularly those with a high refractive index contrast, has increased during the last decade [26,27,28,29,30,31] due to the renewed interest in resonant modes, such as cavity and Fano resonances, and also due to the fact that the nanofabrication of gratings with high aspect ratios has become feasible. In this paper, we discuss the design process of GMR structures using thick grating without PWL for sensing applications, as well as the dependence of the sensors based on a variety of parameters, and we show that it is not possible to perform the design based on planar waveguide theory with homogenized indices for the grating, contrary to the case of thin grating with thin PWL. The experimental performance of a built grating using electron beam lithography as a sensor in the infrared range is presented.

## 2. Application of the Planar Waveguide Sensor Theory of the Thick Grating GMR

**Figure 1:**
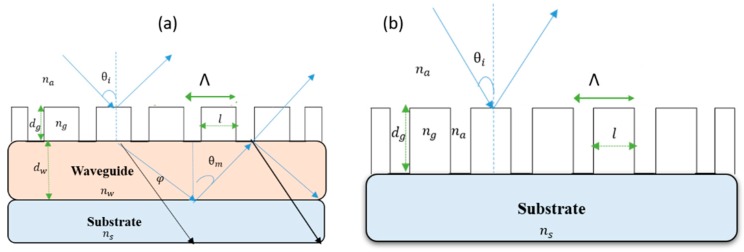
(**a**) Standard guided mode resonance (GMR) structure; (**b**) Thick grating without waveguide layer on substrate.

The geometry of a standard GMR structure is shown in Figure 1a, where the grating layer is on top of the waveguide layer. Figure 1b shows a thick grating structure, which demonstrates resonant reflection modes similar to the regular GMR because the grating layer itself can act as a waveguide. Krasnikov et al. [32] demonstrated that it is possible to predict the GMR sensitivity as a sensor using the Tiefenthaler and Lukosz equations, which describe the phase matching condition for the four layers’ waveguide as it appear in Equation (1) [33,34]:

(1) $$\begin{array}{r} k\left(d_{w}+\Delta d_{g}\right)\sqrt{n_{w}^{2}-n_{eff}^{2}}-arctan\left({\left(\frac{n_{w}}{n_{a}}\right)}^{2\rho }\sqrt{\frac{n_{eff}^{2}-n_{a}^{2}}{n_{w}^{2}-n_{eff}^{2}}}\right)\\ -arctan\left({\left(\frac{n_{w}}{n_{s}}\right)}^{2\rho }\sqrt{\frac{n_{eff}^{2}-n_{s}^{2}}{n_{w}^{2}-n_{eff}^{2}}}\right)≅m\pi \end{array}$$

(2) $$\Delta d_{g}=\left(\frac{n_{G}^{2}-n_{a}^{2}}{n_{w}^{2}-n_{a}^{2}}\right){\left[\frac{{\left(\frac{n_{eff}}{n_{a}}\right)}^{2}-{\left(\frac{n_{eff}}{n_{G}}\right)}^{2}-1}{{\left(\frac{n_{eff}}{n_{a}}\right)}^{2}-{\left(\frac{n_{eff}}{n_{w}}\right)}^{2}-1}\right]}^{\rho }d_{g\;}$$

where ρ = 0 for TE (transverse electric) and ρ = 1 for the TM (transverse magnetic) mode, *n_eff_* is the effective index of the waveguide and the *n_G_* is the effective index of the grating. The refractive indices *n_a_*, *n_w_* and *n_g_* are for the analyte, waveguide layer, and grating material, respectively. The thickness of the waveguide layer is *d_w_* and the thickness of the grating is *d_g_*. For a sub-wavelength grating, where the wavelength is larger than the grating period (*λ* ≫ *Λ*), only the zeroth-order wave propagates while the higher order modes are cutoff. In this case, the grating behaves as a uniaxial birefringent plate, and, therefore, *n_G_* is a function of polarization. Using the nearly quasi-static (NQS) limit, *n_G_* is the solution for two transcendental equations for TE and TM modes [35]:

(3) $$\begin{array}{l} n_{G}{}_{TE}=n_{G}{{}_{TE0}}^{2}+\frac{1}{3}{\left(\frac{\pi f\left(1-f\right)\Lambda }{\lambda }\right)}^{2}{\left(n_{g}^{2}-n_{a}^{2}\right)}^{2}\\ n_{G}{}_{TM0}=n_{G}{{}_{TM0}}^{2}+\frac{1}{3}{\left(\frac{\pi f\left(1-f\right)\Lambda }{\lambda }\right)}^{2}\left(\frac{1}{n_{g}^{2}}-\frac{1}{n_{a}^{2}}\right)n_{G}{{}_{TE0}}^{2}n_{G}{{}_{TM0}}^{6} \end{array}$$

where $n_{G}{}_{TE0}=\sqrt{n_{a}^{2}\left(1-f\right)+fn_{g}^{2}}$, $n_{G}{}_{TM0}=\frac{n_{a}n_{g}}{\sqrt{n_{g}^{2}\left(1-f\right)+fn_{a}^{2}}}$ and f is the fill factor defined as the ratio between the grating line width (*ℓ*) and the period (*Λ*).

Our main interest is to investigate the structure that appears in Figure 1b, using thick grating on a substrate. According to the assumption that the grating will behave as a waveguide, the effective index of the grating replaces the effective index of the waveguide in Equation (1):

(4) $$kd_{g}\sqrt{n_{G}^{2}-n_{eff}^{2}}-arctan\left({\left(\frac{n_{G}}{n_{a}}\right)}^{2\rho }\sqrt{\frac{n_{eff}^{2}-n_{a}^{2}}{n_{G}^{2}-n_{eff}^{2}}}\right)-arctan\left({\left(\frac{n_{G}}{n_{s}}\right)}^{2\rho }\sqrt{\frac{n_{eff}^{2}-n_{s}^{2}}{n_{G}^{2}-n_{eff}^{2}}}\right)≅m\pi .$$

Using the grating equation:

(5) $$n_{a}k\sin\theta_{i}+m\frac{2\pi }{\Lambda }=n_{w}k\sin\theta_{m}\;,$$

where *k* is $\frac{2\pi }{\lambda }$, we can find the effective mode index of the guided mode from $n_{eff}=n_{w}\sin\theta_{m}$ based on a valid approximation for thin grating and a thin waveguide:

(6) $$n_{eff}=n_{a}\sin\theta_{i}+m\frac{\lambda }{\Lambda }$$

where *m* is the diffraction order, and *θ_i_* and *θ_m_* are the incident and the diffraction angles, respectively. The spectral sensitivity appears in Equation (7):

(7) $$\frac{\partial \lambda_{resonance}}{\partial n_{a}}=\frac{\Lambda }{m}\left(\frac{\partial n_{eff}}{\partial n_{a}}-\sin\theta_{i}\right).$$

One of our purposes in this work is to study the effect of different parameters on sensitivity, the location of the resonance, and the width in the IR (Infrared) for the case of thick grating without PWL. 

## 3. Simulation

**Figure 2:**
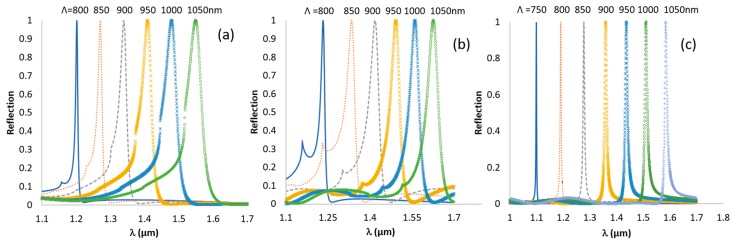
TE (transverse electric) reflection for (**a**) Si_3_N_4_ with *d_g_* = 700 nm and linewidth = 300 nm; (**b**) grating TiO_2_ *d_g_* = 700 nm and space = 700 nm; (**c**) grating SiON_80_ with *d_g_* = 1300 nm and space = 600 nm.

The rigorous simulations of reflectivity were done using the GSOLVER (Grating solver) software, which is an RCWA (Rigorous coupled wave analysis) solver [36]. The refractive indices were taken from the database of GSOLVER. The grating period is affected first, as it has a linear relation with the operation wavelength. The simulation results in Figure 2 are for TE polarization, when the dg and ℓ are fixed and *Λ* is changeable.

The effect of the period is significant and has a linear effect on the location of the resonance. Figure 2 shows that when the period increases, the peak moves to higher wavelengths and the width becomes broader. For normal incidence, and for first order diffraction, Equation (6) reduces to Equation (8), where the relation between the resonance wavelength and the period is linear, at least for the case of thin grating:

(8) $$\lambda_{res}=\;\Lambda n_{eff}.$$

**Figure 3:**
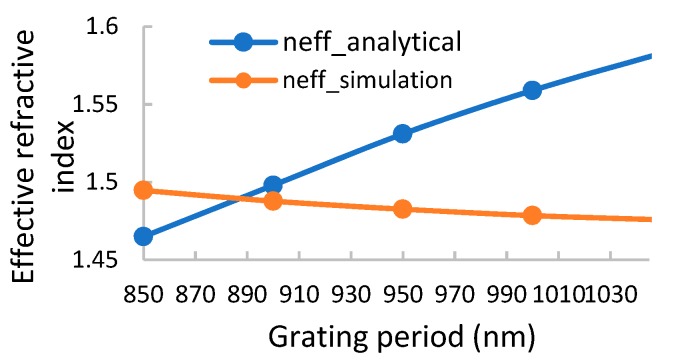
The effective refractive index vs. grating period. For a grating period of 885 nm, the numerical and the analytical refractive index are nearly equal.

From this equation, we can calculate the effective refractive index. In Figure 3, a comparison between the analytical and numerical effective refractive index is shown for Si_3_N_4_ with a thickness of 700 nm and ℓ with a thickness of 300 nm. The numerical refractive index was calculated by using Equation (8), and the analytical refractive index was calculated using the transcendental equation (Equation (4)). Our designs are mainly in the near infrared range because the manufacturability of gratings is easier for this range. In addition, the near infrared range is an optical telecommunication window and therapeutic window, and many optical components, light sources, and detectors are becoming available at low costs. The sensitivity of the sensor is also larger as the wavelength increases.

As seen in Figure 3, the values of the numerical and the analytical effective refractive indexes become close as the grating period decreases, and below 900 nm, they are different by less than 10%. This is expected, as the effective medium approximation is more valid when the wavelength of the period ratio increases for the Rytov approach [32,35]. Although Rytov derived his equations for very thick gratings cases, he did not consider interference effects (when the thickness is larger than a quarter wavelength) and cavity modes. There is a place for further research to derive more accurate expressions under these conditions.

**Figure 4:**
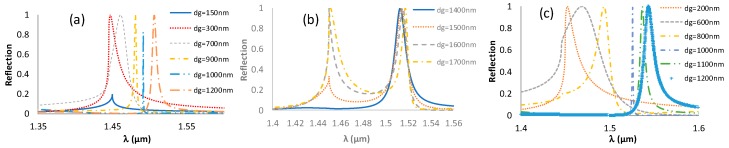
TE reflection from grating with Λ = 1000 nm and *f* = 2/5 made of: SiON_80_ (**a,b**) and Si_3_N_4_ (**c**).

**Figure 5:**
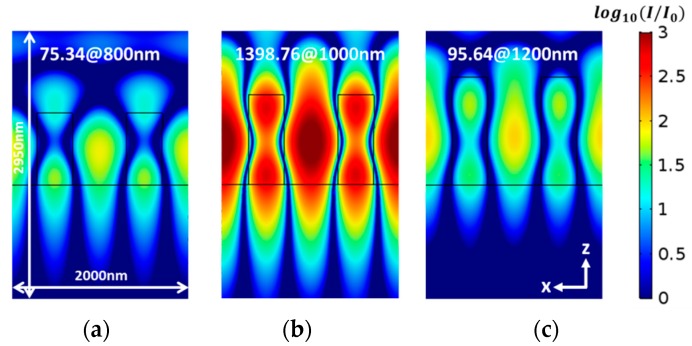
The electric field distribution for grating heights (**a**) 800 nm (at *λ* = 1493 nm); (**b**) 1000 nm (at *λ* = 1525.5 nm); (**c**) 1200 nm (at *λ* = 1544 nm). Note the hot spot value for each case.

The grating height determines the location and width of the signal as it is shown in Figure 4. As the thickness of the grating increases the resonance is shifting to higher wavelengths. Figure 4a,b shows the reflection spectra from SiON_80_, while Figure 4c is for Si_3_N_4_, both with a period of 1000 nm and a fill factor (*f*) of 2/5. In Figure 4b, it is shown that for thickness values greater than 1400 nm, two resonances appear, since higher order guided modes start to be excited. The width of the reflection peak decreases in general with the height of the grating, as expected form waveguide theory. However, at certain grating heights there is a sudden sharp decrease in the width, as it is seen in Figure 4a,c for 1000 nm height. This sudden narrowing of the reflection peak has not been previously reported, to the best of our knowledge (certainly not with the grating/PWL geometry). One might be able to understand this as a result of the excitation of a vertical cavity mode at which the grating lines start to act as dielectric optical antenna. To verify this, we performed field distribution calculations for different grating thicknesses around 1000 nm. A comparison of the field distribution for Si_3_N_4_ grating with different heights at a fill factor of 2/5 was calculated using the COMSOL software, a finite element solver; the package features an RF model/wave optics model. The beam size is infinite, although in reality it is always finite. However it is well known that after a beam diameter of around 20 periods, the size effects are negligible. For a height of 1000 nm, the peak becomes narrow and the field enhancement increases by a factor of 18.56, as shown in Figure 5. The field pattern has one strong maximum in the center of the spacing between the lines, and inside the lines it has two maxima, one near the top and one near the bottom. This pattern reminds us of nanoantenna behavior, which occurs when its length equals a multiple number of effective half waves. On one hand, the effective index satisfies Equation (8). However, because the thickness of 1000 nm is also equal to the period, we can state that Equation (8), with the period replaced by the thickness, is satisfied. This is verified by the fact that in Figure 4a,c, similar ultra-narrow peaks are obtained when the period equals the thickness. Hence, the condition to get this ultra-narrow peak seems to be that the thickness equals the period. However this point needs further verification, which is planned in the near future. A narrow peak was also observed in pervious works [37], where the thickness conditions for symmetrical line shape is equal to half the resonance wavelength (*d* = λ_resonance_/2) for a symmetrical structure. However, in this work, the results did not show a sudden narrowing for a specific grating thickness. Further study is planned for this intriguing high Q factor resonance, perhaps in light of modern theories for high contrast gratings [38] and other dielectric nano-antenna theories. The fact that the field is enhanced drastically at this narrow resonance has important implications not only for RI sensing but also to enhance spectroscopic signals, such as Raman and fluorescence.

**Figure 6:**
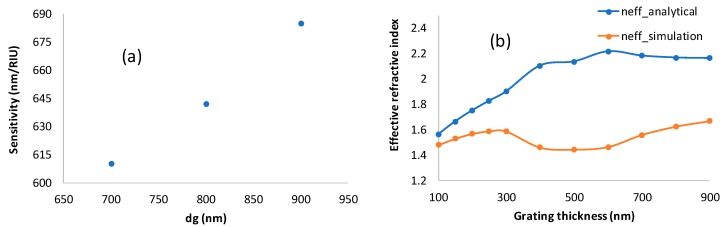
(**a**) The sensitivity for TiO_2_ grating with Λ = 1000 nm and *f* = 3/10; (**b**) The effective refractive index vs. grating thickness for TiO_2_ grating with space equals to 600 nm.

The relation between the thickness and the sensitivity for TiO_2_ is shown in Figure 6a. As the thickness of the grating becomes higher, the sensitivity increases as one might expect, since the grating spaces are filled with the analyte. However, the difference between the analytical refractive index and the simulated refractive index becomes smaller when the height of the grating decreases, as shown in Figure 6b. The reason for this occurrence is because the effective medium approximation is more valid for thinner gratings. An effective medium theory valid for thick gratings does not exist, particularly when resonances exist in spite of the attempts of some investigators to predict analytically the depth dependence of the effective medium approach [39] for thin gratings.

**Figure 7:**
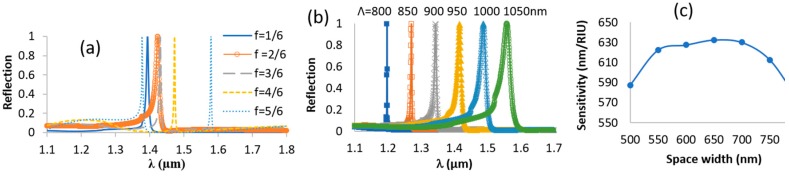
TE Reflection spectra from Si_3_N_4_ grating with *d_g_* = 800 nm (**a**) with a constant period (Λ = 950 nm), and *f* is increasing; (**b**) a constant fill factor (*f* = 1/4) and a different period. (**c**) Sensitivity for Si_3_N_4_ grating, *Λ*= 1000 nm and *d_g_* = 800 nm.

The third variable is the fill factor, and for this, two cases were tested. Figure 7a shows the effect where the period is constant and the fill factor increases. In the latter case, the resonance shifts to longer wavelengths because the effective index increases with the fill factor. At a high fill factor, a higher order mode also appears. Figure 7b shows the case when the fill factor is fixed but the period is changing. As expected in this case, the resonance shifts linearly with the period similar to what is presented in Figure 2. The sensitivity for Si_3_N_4_ grating and a fill factor of 1/4 with a height of 200 nm and 800 nm is approximately 45 nm/RIU and 570 nm/RIU, respectively, demonstrating the strong effect of the grating height on the sensitivity when the spaces are empty. The optimal sensitivity for Si_3_N_4_ grating is when the space width is 650 nm, as shown in Figure 7c, which is also expected because, above a certain space, the field strength at the midway between the two adjacent lines becomes weaker. 

**Figure 8:**
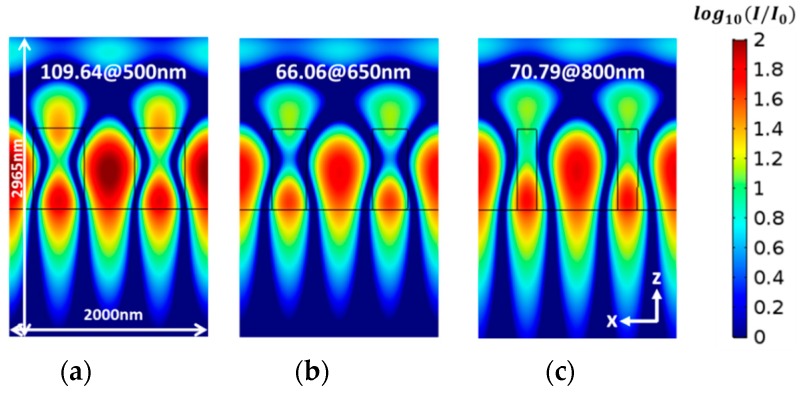
The intensity distribution of the electric field for different grating spaces with Λ = 1000 nm and *dg* = 800 nm; (**a**) grating space of 500 nm at *λ* = 1500 nm; the maximum intensity is 109.64; (**b**) grating space of 650 nm at *λ* = 1492 nm, the maximum intensity is 66.06; (**c**) a grating space of 800 nm at *λ* = 1476 nm; the maximum intensity is 70.79.

In an attempt to correlate the field enhancement and the sensitivity, we calculated the field distribution intensity for different space widths, as shown in Figure 8. Although some variations are seen, there is no clear correlation between the field distribution and the sensitivity. The reason for this result is perhaps because the variations in the sensitivity, as seen in Figure 7c, are as small as the space width varies.

**Figure 9:**
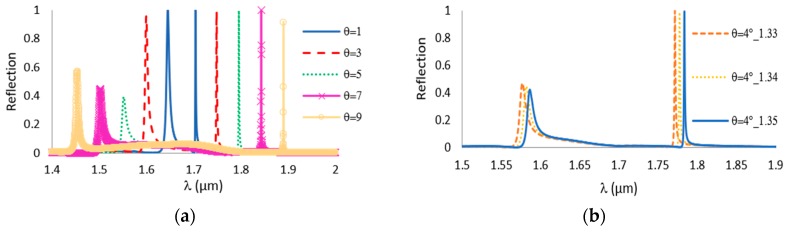
TE reflection spectra SiON80 grating with *dg* = 1300 nm, Λ = 1100 nm, and *f* = 9/22.

The influence of the oblique incidence angle is shown in Figure 9a. The parameters for SiON_80_ grating are as follows: *f* = 9/22, Λ = 1100 nm and *d_g_* = 1300 nm. For the oblique angle, two peaks were generated. This is expected, as the +1st and −1st order harmonic waves are now at different angles. As the incidence angle increases, the first peak moves to longer wavelengths, and the second peak moves to shorter wavelengths. The width of the second peak is wider than the first peak. The location of the peaks has a linear relation with the incidence angle following Equation (6). As it appears in Figure 9b, the change in the refractive index of the analyte causes both peaks to move. At an incidence angle of 4°, the sensitivity of the first peak and second peak are 600 (nm/RIU) and 470 (nm/RIU), respectively. This is also expected, as the sensitivity changes almost linearly with the wavelength if we ignore the dispersion.

**Figure 10:**
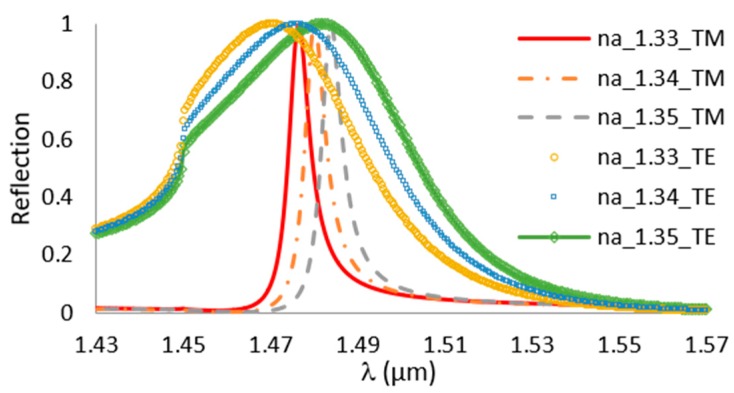
The reflection for Si_3_N_4_ grating for TE and TM polarization, *dg* = 600 nm, Λ = 1000 nm and *f* = 2/5.

The polarization has another effect on the location of the peak and the width of the signal. Figure 10 compares between two polarizations, TE and TM, for Si_3_N_4_ grating with *f* = 2/5, a period of 1000 nm, and a height of 600 nm. The Sensitivity of TE polarization is 575 (nm/RIU), and for the TM polarization, it is 375 (nm/RIU). Note that the TE polarization peak is also much wider than that of the the TM. The width of the peak expresses how well defined the guided mode is. The more it is confined, the larger the part of its energy is in the waveguide (within the grating region), and, due to the interaction with the analyte in the spaces region, a large sensitivity is obtained. On the other hand, when the mode has most of its energy outside the grating region, the effect of the analyte index changes on the mode are less.

The figure of merit (FOM) is the ratio between the sensitivity and the FWHM (full width half maximum), which defines the detection limit. For the TE and TM polarization, the FOM is approximately 11 and 68, respectively. The variations of the TE and TM resonances with the different parameters becomes extremely complicated as the grating height increases, as many other phenomena start to take place simultaneously (in addition to the wave guiding), such as interference, cavity modes, and resonant nanoantenna. For example, the inflection type point in the shape of the TE mode near 1.44 μm can be a result of these different phenomena.

Table 1 compares different properties for GMR without a waveguide and GMR with a waveguide. Some of the properties are similar for both sensors.

## 4. Experimental Details and Results

**Figure 11:**
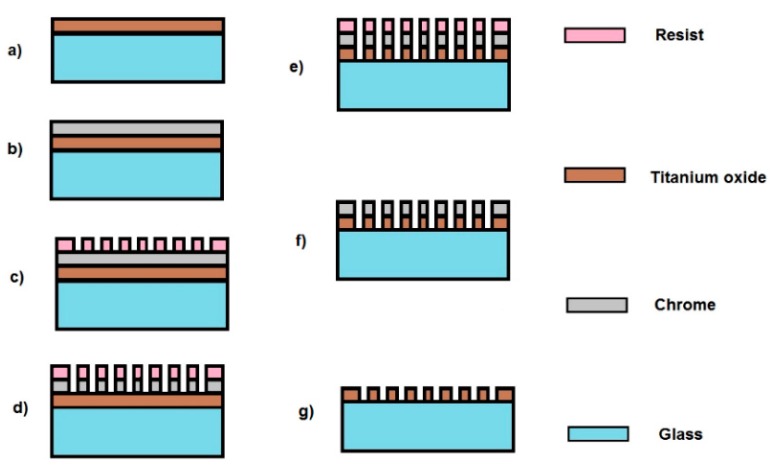
Process flow schematic of TiO_2_ grating fabrication: (**a**) TiO_2_ sputtering; (**b**) Cr deposition; (**c**) e-beam lithography on resist; (**d**) Cr dry etch; (**e**) TiO_2_ dry etching; (**f**) resist removing; and (**g**) Cr wet etch.

**Figure 12:**
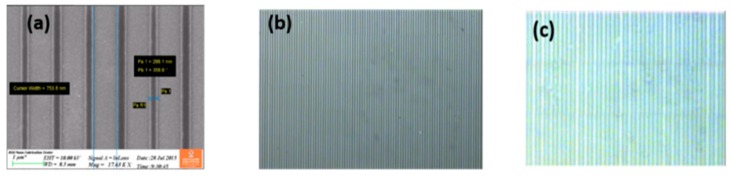
SEM (Scanning electron microscope) images of the grating lines at different stages: (**a**) After e-line lithography; (**b**) Chrome etched in the pattern; and (**c**) TiO_2_ and Chromium layer in pattern etched in DRIE (Deep reactive-ion etching).

The TiO_2_-on-glass grating was prepared by etching in fluorine containing ICP (inductively coupled plasma). 600 nm TiO_2_ film and 70 nm of metallic chrome were sputtered on the glass substrate, as shown in the process flow schematic below (Figure 11a,b). The chrome film was then lithographically patterned using an e-beam according to the desired grating and etched through by chlorine containing plasma. Thus, a hard lithographic mask was obtained for the followed titanium oxide etching. Figure 12 shows scanning electron microscope (SEM) images for different stages during the fabrication of the grating. 

**Figure 13:**
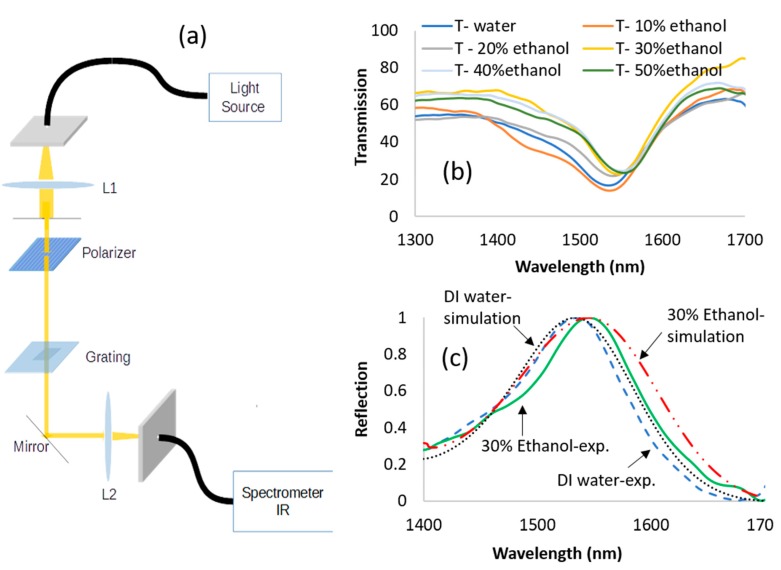
(**a**) Transmission setup; (**b**) transmission spectrum at the TE polarization; (**c**) comparison between the spectrums of the simulation and the experiment.

**Figure 14:**
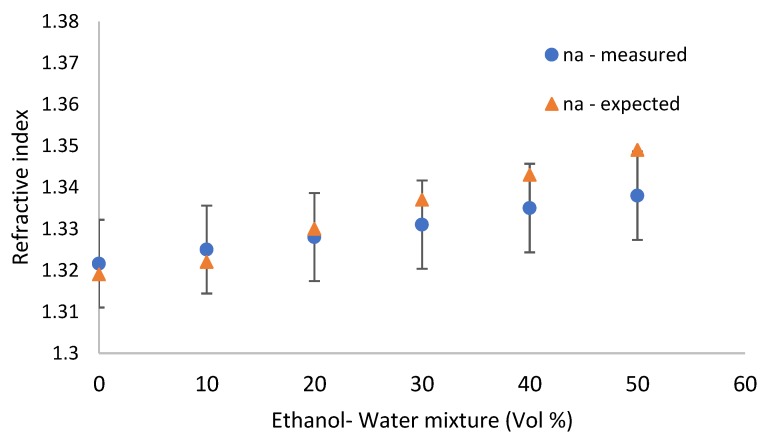
Comparison of the measured and expected refractive indices.

Using transmission setup as shown in Figure 13a, different mixtures of ethanol and water were checked in the infrared range (1.4 µm–1.7 µm) as it appears in Figure 13b, where *n_a_* is the refractive index of the analyte. Knowing the grating parameters and the measured peak wavelength, the refractive indices of the analyte were estimated from the simulations in Figure 13c and compared to the expected ones for the water–ethanol mixtures [43], as shown in Figure 14. The sensitivity of the manufactured grating is 658 nm/RIU, and the resolution is 0.06 nm, while in the simulation, the sensitivity is 680 nm/RIU. A sensitivity of 418 nm/RIU with a high Q factor resonance was reported for the silicon grating [44]. A good fit is observed between the experimental and the theoretical curves. Our design was to obtain a resonant peak with a few tens of nm in width in order to minimize the effect of manufacturing non-idealities on the peak height, because the manufacturability of deep gratings is prone to deviations from the ideal structure when E-beam lithography is used. The manufacturing non-idealities caused the peak to be wider in the tens of nm range. However, it did not affect its height as much. In the future, we plan to manufacture larger area gratings with narrower widths using nanoimprinting and deep UV lithography, as is being done in semiconductor manufacturing foundries. Our main purpose of highlighting the potential of a thick resonant grating structure for sensing and optical filtering is achieved here, both theoretically and experimentally. Thermal effects should be considered on a real word sensor. This can be avoided by keeping the temperature constant using temperature control and a thermoelectric cooler. One can also add a reference channel containing a liquid with a known thermooptic coefficient, such as DI (distilled water) water and use a pre-calibration procedure, as with some commercially available biosensing instruments [45,46].

## 5. Conclusions

A GMR structure that consists of thick grating without a waveguide layer was investigated both theoretically and experimentally. Different parameters, such as the height of the grating, the grating period, and the fill factor have an effect on the sensitivity, the location of the resonance, and the width of the peak. Different dielectric materials were checked: TiO_2_, SiON_80_, and Si_3_N_4_. A TiO_2_ grating with a period of 1050 nm, thickness of 650 nm, and grating linewidth of 300 nm was manufactured with e-beam lithography and tested in transmission mode in the SWIR range, showing reasonable agreement with the theory. Compared to the GMR structure with PWL, the thick grating-only GMR structure has several unique properties: (i) It gives higher sensitivity when the spaces are filled with the analyte, peaking at a certain space value due to an increase in the interaction volume between the analyte and the evanescent optical field between the grating lines; (ii) the TM resonance in certain cases gives a better figure of merit; (iii) the sensitivity increases as the grating height increases; (iv) the prediction of the resonance locations based on the effective medium approximation does not give satisfactory results when the grating height is larger than a certain value and the invalidity becomes more severe as the period increases. Unlike the case of thin grating with waveguide, in the thick grating case, the effective index decreases with the period based on rigorous calculations; (v) a sudden drop in the peak width occurs at a specific height value, and at this value, the field distribution exhibits ultrahigh field enhancement; (vi) it simplifies the production of GMR sensors, as its tolerances in the waveguide layer parameters are usually tough. The high local field enhancement may be used to enhance the local plasmonic field near metal nanoparticles by coupling between this special mode and the local plasmons [47,48] to further enhance spectroscopic signals in parallel to refractive index sensing. In addition, apart from the guided mode resonance, thick dielectric [49] and metallic gratings [50] are known to reveal many other resonances and rich spectra that are sensitive to the analyte refractive index, and the whole spectrum can be used to detect refractive index variations. The manufacture of thick grating-based sensors that give narrower resonances is becoming more feasible, a fact that improves the figure of merit and so also the resolution.

## Figures and Tables

**Table 1:** Comparison of the properties of the GMR sensors. FWHM, full width half maximum.

Property	GMR without a Planar Waveguide	GMR with a Planar Waveguide
Location of the resonance as a function of grating thickness	As the thickness of the grating increase the resonance shifts to longer wavelength.	
Location of the resonance as a function of grating period	As the period increases the resonance shifts the longer wavelength.	
FWHM as a function of polarization	For TM the FWHM is smaller	
Typical sensitivity in the angular configuration	70°/deg	35.8°/deg [40]
Sensitivity as a function of fill factor (*f*)	For *f* between 0.25–0.5 the sensitivity is maximum.	As the f increase the sensitivity decreases. [41]
Sensitivity as a function of thickness	As the thickness of the grating increases, the sensitivity increases.	As the thickness of the waveguide increases the sensitivity decreases. [42]
